# The Effect of Different Types of Musculoskeletal Injuries on Blood Concentration of Serum Amyloid A in Thoroughbred Racehorses

**DOI:** 10.1371/journal.pone.0140673

**Published:** 2015-10-14

**Authors:** Agnieszka Turło, Anna Cywińska, Michał Czopowicz, Lucjan Witkowski, Artur Niedźwiedź, Malwina Słowikowska, Hieronim Borowicz, Anna Jaśkiewicz, Anna Winnicka

**Affiliations:** 1 Department of Pathology and Veterinary Diagnostics, Faculty of Veterinary Medicine, Warsaw University of Life Sciences–SGGW, Warsaw, Poland; 2 Laboratory of Veterinary Epidemiology and Economics, Faculty of Veterinary Medicine, Warsaw University of Life Sciences–SGGW, Warsaw, Poland; 3 Department of Internal Diseases with Clinic for Horses, Dogs and Cats, Faculty of Veterinary Medicine, Wrocław University of Environmental and Life Sciences, Wrocław, Poland; Texas Tech University Health Science Centers, UNITED STATES

## Abstract

**Background:**

Training-induced muscle, skeletal and joint trauma may result in acute phase response reflected by the changes in the blood concentration of serum amyloid A (SAA) in racehorses. It remains yet unclear if such systemic reaction could be triggered by sport injuries and what is the impact of different types of musculoskeletal trauma on SAA concentrations in racehorses. This study aimed to determine changes in the SAA blood concentration in racehorses with different types of injuries of musculoskeletal system.

**Materials and Methods:**

The study involved 28 racehorses diagnosed after the race with bone fractures (n = 7), dorsal metacarpal disease (n = 11), joint trauma (n = 4) or tendon and muscle trauma (n = 6) and 28 healthy control racehorses. Serum samples were collected twice, between 1 and 4 days of the injury or succesful completion of the race. SAA concentration was measured using the commercial ELISA kit. Differences between mean SAA concentration in respective groups were analyzed using ANOVA and Tukey post-hoc test.

**Results:**

Mean SAA concentration within the first 4 days of the injury of muscle and tendon was significantly higher than in bone fractures, dorsal metacarpal disease, joint trauma or in the healthy horses (p<0,001). There were no significant differences between the other groups.

**Conclusions:**

Strain injuries of muscle and tendons can cause a moderate increase in SAA blood concentration in racehorses, reflecting the occurrence of the acute phase response. Similar reaction is not observed in the stress-related bone injuries.

## Introduction

Serum amyloid A (SAA) is the main acute phase protein (APP) in horses, massively released into the bloodstream in the initial phase of inflammation, known as the acute phase response (APR). Elevated blood concentration of the positive APPs is one of the main effects of the APR. Their production in the liver is mediated by the proinflammatory cytokines–interleukin 1 (IL-1), interleukin 6 (IL-6) and tumor necrosis factor α (TNFα), secreted by the phagocytic cells in response to various types of tissue damage [1]. Due to the fast and intensive reaction to cytokine stimulation, the main APPs are considered sensitive biomarkers of the diseases accompanied by systemic inflammation [2,3]. In horses affected by inflammatory disorders of gastrointestinal, respiratory and reproductive system or developing complications after surgical procedures, SAA level can exceed the physiological range from 10 to 1000 times and may be useful in disease diagnostics and prognostication [3–8].Much less is known about the relation between the APPs and disorders of the musculoskeletal system. Injuries of bone, muscle and tendon resulting from repetitive mechanical overload are the main health issues in performance horses. In racehorses they are responsible for the greatest number of the lost training days [9]. Early recognition of horses on risk of suffering the injury is crucial and the range of blood biomarkers have been examined in that context [10–12].

We previously reported that racehorses with stress-related injuries of musculoskeletal system showed higher SAA levels than the non-injured horses in the 3^rd^-4^th^ day after the race [13]. The examined group included horses with injuries of different location and pathogenesis and large individual variations in SAA concentration were observed. Our hypothesis is that the ability of the stress-related musculoskeletal injuries to induce the APR, and thus increase the SAA level, could vary depending on the type of the injury and the tissues involved. Therefore, we aimed to compare changes in SAA blood concentration among racehorses diagnosed with different types of stress-related injuries of musculoskeletal system and healthy racehorses subjected to exercise. Unlike most of the reports on naturally occuring injuries, this study analyzed SAA level with regard to the time of acquisition of the injury. The group of injuries of musculoskeletal system associated with the SAA reaction was identified.

## Materials and Methods

### Horses and injuries

This case-control study involved 56 Thoroughbred racehorses aged 2 to 7 years (median age of 2 years, interquartile range from 2 to 3 years), stabled and regularly trained in different horse racing facilities in Poland. This study was approved by the III Local Ethical Committee for Animal Experiments at Warsaw University of Life Sciences–SGGW (permit number: 68/2013), the trainers and the owners of the horses.

The experimental group consisted of 28 racehorses (median age– 2 years, interquartile range from 2 to 3 years) enrolled on the basis of the clinical signs of acute musculoskeletal injury (lameness, heat, swelling, sensitivity to palpation) observed after the race or intensive training session (breezing), which led to suspension of training for the minimum of 7 consecutive days. Diagnosis of the injuries was based on clinical examination and diagnostic imaging (radiographs and ultrasound) performed by the equine veterinary practitioner. In the days following the injury horses were subjected to stall rest or light exercise (30 min in horse walker) depending on the veterinary recommendations. According to the diagnosis, horses were divided into four groups: bone fractures (n = 7), dorsal metacarpal disease (n = 11), joint trauma (aseptic arthritis) (n = 4) and muscle and tendon trauma (n = 6).

### Fractures

All fracture cases included in the study were closed, of nontraumatic aetiology and manifested after intensive exercise. Accordingly, they were classified as stress fractures. The fractures were confirmed by radiography or ultrasound and located in the distal limb (the carpus and lower bones).

### Dorsal Metacarpal Disease (DMD)

The cases of DMD were primarily identified by trainers, who recognized acute lameness, heat, sensitivity to palpation and swelling at the dorsomedial surface of the metacarpus in one or both front legs. Diagnostic imaging was requested by trainers in single cases only–therefore, the inclusion criteria in this group involved, besides the clinical manifestation of DMD, missing at least 7 days of regular training due to the condition.

### Aseptic arthritis (joint trauma)

Horses allocated in the aseptic arthritis group were identified with unilateral or bilateral joint effusion associated with pain, lameness or reluctance to train, and did not show any bone or tendon abnormalities in radiographical and ultrasound examination. The trainers of the horses refused to perform the analysis of the synovial fluid, however, the acute synovitis emerging after intensive training, lack of radiological changes and remission of clinical signs within several days of rest substantiated the diagnosis of stress-related aseptic arthritis.

### Muscle and tendon injury

Cases in this group included tendonitis of the front SDFT, confirmed in the ultrasound examination by the enlargement of the cross-sectional area of the tendon compared with the contralateral leg. Muscle injury was represented by exertional rhabdomyolysis (ER), manifested by muscle pain and firmness developing short after the beginning of exercise and confirmed by the high creatinine kinase and aspartate aminotransferase blood levels.

The control group included 28 healthy Thoroughbred horses (median age– 2 years, interquartile range from 2 to 3 years) that did not have any hematological abnormalities and lacked clinical signs of the injury after completion of the race and in the following days. Control horses were all subjected to the light exercise in horse walker in the sampling period due to the end of the racing season. Hematology control was performed in all horses in order to minimize the possible effect of concomitant health issues other than musculoskeletal injury and as a general health control in the non-injured horses. Racing and training distances for all horses varied from 1200 to 1600 m, accordingly to their age and experience and depended on the horse’s trainer.

### Blood samples

Blood was collected from injured horses on the 1^st^ (16 horses) or 3^rd^ (12 horses) day after the injury depending when the injury was notified by an owner. Exact time of blood collection is presented in Table 1. Control horses were blood-sampled on both 1^st^ and 3^rd^ day after the race. All blood samples were acquired by a jugular venipuncture into 20 ml syringes and transferred immediately into K2-EDTA tubes for hematological tests and plain tubes for SAA analysis. EDTA blood samples were kept in +4°C and examined within 5 h for routine hematological parameters in an automated analyzer (ABC Vet, Horiba ABX). Plain tubes were centrifuged at 4380g for 5 minutes, collected serum was frozen and stored at -20°C for SAA analysis. SAA concentrations were measured using an enzyme linked immunosorbent assay (PHASE SAA Assay, Tridelta Ltd) previously validated for use in equine studies [13–17]. Basic dilution of the samples was 1:1000. Samples with SAA values above the detection limit were further analyzed at the dilution of 1:2000.

**Table 1:** Number of injured horses blood sampled on the 1^st^ and 3^rd^ day.

Injury type	1^st^ day	3^rd^ day
Bone fracture (n = 7)	3	4
Dorsal metacarpal disease (n = 11)	7	4
Joint trauma (n = 4)	3	1
Tendon/muscle trauma (n = 6)	3	3
Total (n = 28)	16	12

### Statistical analysis

Two-factor analysis of variance (ANOVA) including two categorical variables as fixed effects–type of injury (5 categories– 4 injuries and a control) and a day of blood collection (2 categories– 1^st^ or 3^rd^ day)–was performed to control for various days of blood collection. To cope with unbalanced study design (unequal replications of categories in 5x2 table) and achieve at least proportional replication of categories [18] 28 control horses were randomly split in two groups of 16 and 12 horses and blood samples collected from them on the 1^st^ or 3^rd^ day, respectively, were included in the analysis. As data were non-normally distributed according to a Shapiro-Wilk test (p<0.001 for all horses included; strong right-hand skewness–Pearson’s coefficient of skewness = 2.43) as well as heterogeneity of variances was present among groups according to a Levene’s test (p<0.001), natural logarithmic transformation was applied to satisfy assumptions of a two-factor ANOVA (after transformation: Shapiro-Wilk test p = 0.065 for dorsal metacarpal disease to 0.999 for joint trauma, Pearson’s coefficient of skewness = -0.02; Levene’s test p = 0.073). A Tukey test for unequal samples was used in post-hoc analysis. A p-value below 0.05 was considered to indicate statistical significance. Statistical analysis was carried out in Statistica 10 (StatSoft Inc.). For the needs of reporting results transformed units were converted back to the original ones and SAA concentration was given as median, interquartile range (IQR) and range. Morphological parameters, normally distributed according to a Shapiro-Wilk test were presented as the arithmetic mean ± standard deviation.

## Results

**Fig 1:**
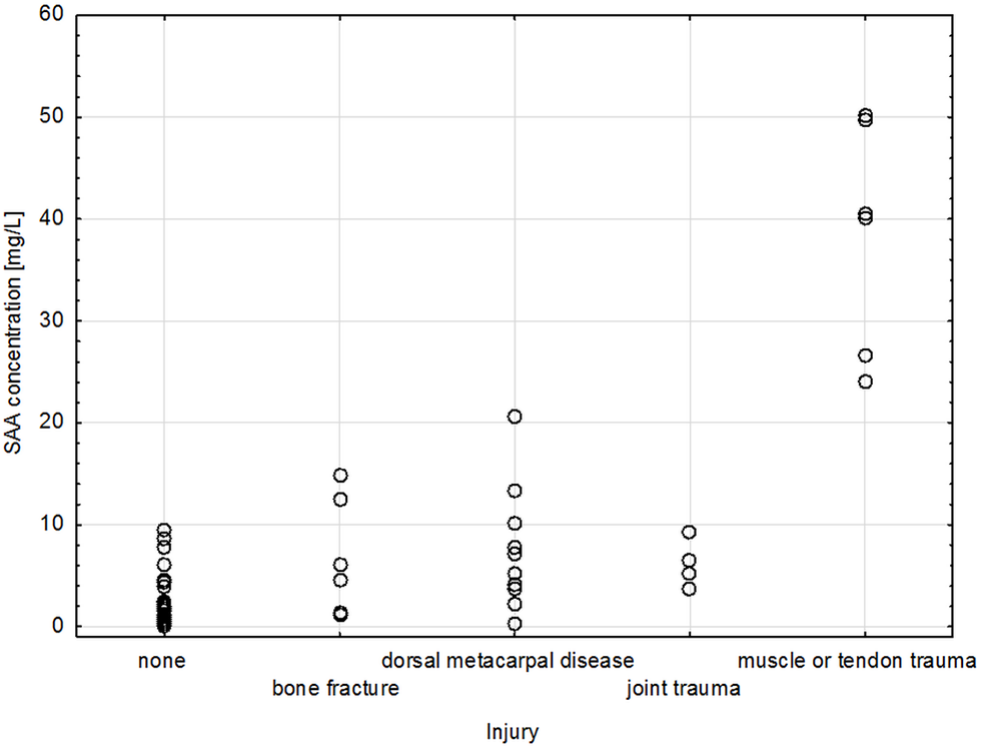
SAA concentration in five groups of horses. * SAA concentration significantly higher than in the rest of groups (p<0.001).

Analysis with two-factor ANOVA indicated a significant difference between types of injuries. Moreover, interaction between a type of injury and a day of blood collection was significant (Table 2). Having taken a day of blood collection into account the mean SAA concentration proved significantly higher in horses with tendon/muscle trauma compared to horses with other injuries (p<0.001). No significant differences were observed between other types of injury (Table 3, Fig 1). All hematological parameters in all horses remained within the reference ranges for Thoroughbred horses [19] (Table 4).

**Table 2:** Results of two-factor ANOVA.

Source of variation	df	Mean square	F	p
Type of injury	4	13.49	16.35	<0.001
Day after the race	1	3.03	3.67	0.062
Interaction	4	2.43	2.94	0.030
Error	46	0.83	-	-

**Table 3:** Serum amyloid A concentration in different types of injuries and in healthy horses.

Injury type	SAA concentration [mg/L]
Control (n = 28)	1.73 (0.87–3.25; 0.17–9.44)
Bone fracture (n = 7)	4.66 (1.23–12.45; 1.08–14.85)
Dorsal metacarpal disease (n = 11)	5.23 (2.30–10.06; 0.22–20.61)
Joint trauma (n = 4)	5.96 (4.51–7.95; 3.70–9.29)
Tendon/muscle trauma (n = 6)	40.22 (26.65–50.00; 24.11–50.00)*

Median (IQR; range)

* SAA concentration significantly higher than in the rest of groups (p<0.001)

**Table 4:** Hematological parameters in the injured and the control horses.

Injury type	WBC (G/L)	RBC (T/L)	HCT (%)	HGB (g/dL)
Control (n = 28)	6.83±1.54	10.19±1.02	43.90±4.23	14.50±1.37
Bone fracture (n = 7)	8.26±1.60	10.51±1.29	44.63±5.54	13.40±2.52
Dorsal metacarpal disease (n = 12)	9.35±1.79	9.54±1.37	40.19±6.28	11.58±3.10
Joint trauma (n = 4)	9.57±1.73	9.44±0.87	38,56±4.72	11.54±2.22
Tendon/muscle trauma (n = 6)	9.29±3.58	9.96±1.23	43.36±10.45	14.50±3.12

Mean ± standard deviation

WBC–white blood cell count, RBC–red blood cell count, HCT–packed cell volume, HGB–hemoglobin concentration

## Discussion

Distribution of the injury types within the examined group was typical for the stress-related musculoskeletal lesions in young Thoroughbred racehorses [20]. Our results have shown that SDFT tendonitis and muscle injury, represented in this study by the exertional rhabdomyolysis (ER), are the only injuries of the locomotor system that result in SAA elevation when compared with the control group [3]. Moreover, SAA levels in this group reached the values that are considered indicative for systemic reaction in horses with inflammatory diseases (50 mg/l) [3,21]. ER has been previously reported to cause the radical increase in SAA serum concentration in Arabian horses [17]. Our study included one case of ER that showed the highest SAA value among all of the examined horses (50 mg/l), although lower than the levels described by El-Deeb et al. [17]. This single result does not allow to hypothesize about the influence of breed or type of effort on the SAA level in horses with ER, nevertheless, it confirms the systemic effect of this muscle disorder. Another study by the same research team [22] indicated the significant increase in serum IL-6, TNF-α and PGF2-α in horses affected with ER. IL-6 is the main cytokine produced by the contracting skeletal muscle [23–25]. The release of IL-6 from muscle during exercise in human was suggested to reach sufficient intensity in order to account for its noticeable accumulation in blood [24]. Numerous studies have shown significant increase of IL-6 and other proinflammatory cytokines (IL-1β, TNF-α, interferon γ - INF-γ) in blood in human and horses after intensive or prolonged exercise [25–31]. Several authors proposed the stress-induced damage of muscle fibers as the main cause of blood cytokine increase after exercise, supported by the positive correlation between cytokines and muscle enzyme concentrations or the delayed onset muscle soreness (DOMS) [22,29,32]. Whereas direct link between exercise-related muscle damage and the induction of the APR still needs to be elucidated, the evidence of local cytokine production in muscle that leads to the systemic response may facilitate search for similar mechanism in other tissues of the musculoskeletal system.

The endogenous production of proinflammatory cytokines in tenocytes stressed by application of cyclical strain, hypoxia and in tendon injuries is well documented [33–37]. Increased experession of IL-1β, IL-6, TNFα, interleukin 8 (IL-8) and monocyte chemoattractant protein 1 (MCP-1) was observed in rodent and rabbit models of tendon damage, human tenocyte culture and in the injured tendon samples. The only available equine study showed positive immunohistochemical staining for IL-1, TNF-α and INF-γ in the fragments of the inflamed SDFT, although it did not evaluate the IL-6 production [37]. While the auto- and paracrine effects of cytokines in tendon tissue and their role in injury healing is being investigated, it is still unknown to what extent the locally produced mediators of inflammation affect the general homeostasis. Langberg et al. compared the IL-6 concentrations measured simultanously in plasma, muscle and peritendinous tissue in the human long-distance runners [38]. The results demonstrated that interstitial concentration of IL-6 in connective tissue surrounding the Achilles tendon was approximately 10-fold higher than collected from muscle and corresponded in time with the increase of IL-6 level in plasma. In our study tendonitis of the SDFT, the structure functionally and clinically equivalent to the human Achilles tendon, resulted in the elevated SAA blood level. This finding appears to corroborate the hypothesis that local inflammatory response within the connective tissue of the tendon, can reach the blood threshold necessary to induce the APR. Further investigation of this relationship is warranted.

There are several reports on the SAA response in serum and synovial fluid in horses with naturally acquired or experimental joint disease [39–41]. In the clinical cases of joint disease, SAA concentration tends to increase in horses with an infectious arthritis or suspected of the bacterial contamination of the joint but remains low in the non-infectious joint disorders [41]. Our study included four horses diagnosed with the aseptic, stress-related arthritis, typically encountered in young racehorses introduced to the high-intensity training. None of the affected horses showed SAA serum level higher than the control group, presumabely due to the mild character of joint inflammation (limited to the synovial membrane of the joint capsule) and lack of the infectious agent.

Cases of bone injuries enrolled in this study showed different degree of severity, however, all of the fractures were categorised as closed, stress-related lesions of the distal limb accompanied with a limited soft tissue damage. Four of them were intraarticular fractures located in the carpus. Neither bone fractures nor periostitis (DMD) resulted in the marked APR, unlike the bone injuries in human and mice [42–44]. To authors’ best knowledge, there are few studies on this subject referring to horses. Occurrence of the intraarticular fracture in horses caused the radical increase of IL-6 and TNF-α in the synovial fluid in contrast to the osteoarthritis not associated with fracture [45,46]. The early work on the serum concentration of the moderate APP, fibrinogen, did not show any abnormalities in horses with fractures of the distal leg–however, the authors of did not report the time from the injury to the blood collection, so it may be suspected that it was not sufficiently long for fibrinogen to reach it’s peak concentration [3,47]. In the recent study we tested the SAA level in the 1^st^ and 3^rd^ day after injury. In this time SAA has been reported to reach it’s highest concentration in the course of the APR [3,8,13,16,39,40,48], therefore, it is unlikely that the SAA reaction in any of the groups in this study was overlooked. The lack of SAA release from the liver in horses with distal bone fracture may suggest that the release of IL-6 on the site of injury is limited compared to the injuries of the soft tissues and as a result, the concentration of this cytokine in blood is too low to stimulate the systemic reaction and SAA synthesis. In stress fractures of the distal limb the mass of the inflamed tissues is smaller than in the injuries of tendon and muscle. Together with the relatively poor vascularization of the bone, it could affect the secretion of locally producted cytokines into the blood, resulting in decreased attraction of the leucocytes to the site of injury and more restrained systemic reaction.

In conclusion, injuries of the soft tissue structures within musculoskeletal system may result in the increased serum SAA concentration in Thoroughbred racehorses, high enough to be interpreted as the APR, while damage to the bone and cartilage does not induce the systemic reaction. On the basis of this observation we cannot determine the cause of this difference, although lower mass of the affected hard tissues and their reduced vasculature may be a limiting factor for the release of locally produced proinflammatory mediators, mainly IL-6, to the blood stream. Injuries of muscle and tendon related to mechanical stress associated with race training can affect the serum level of SAA and should be considered in the interpretation of this biomarker in racehorses.

## References

[1] Gabay C, Kushner I. Acute-phase proteins and other systemic responses to inflammation. N Engl J Med. 1999;340: 448–454. 9971870 10.1056/NEJM199902113400607

[2] Cray C. Acute phase proteins in animals 1st ed. Progress in Molecular Biology and Translational Science. Elsevier Inc.; 2012. 10.1016/B978-0-12-394596-9.00005-6 PMC7149966 22137431

[3] Jacobsen S, Andersen PH. The acute phase protein serum amyloid A (SAA) as a marker of inflammation in horses. Equine Vet Educ. 2007;19: 38–46.

[4] Belgrave RL, Dickey MM, Arheart KL, Cray C. Assessment of serum amyloid A testing of horses and its clinical application in a specialized equine practice. J Am Vet Med Assoc. 2013;243: 113–9. 10.2460/javma.243.1.113 23786199

[5] Canisso IF, Ball BA, Cray C, Williams NM, Scoggin KE, Davolli GM, et al Serum amyloid A and haptoglobin concentrations are increased in plasma of mares with ascending placentitis in the absence of changes in peripheral leukocyte counts or fibrinogen concentration. Am J Reprod Immunol. 2014;72: 378–385. 10.1111/aji.12278 24916762

[6] Pihl TH, Scheepers E, Sanz M, Goddard A, Page P, Toft N, et al Influence of disease process and duration on acute phase proteins in serum and peritoneal fluid of horses with colic. J Vet Intern Med. 2015;29: 651–658. 10.1111/jvim.12542 25644457 PMC4895517

[7] Jacobsen S, Jensen JC, Frei S, Jensen AL, Thoefner MB. Use of serum amyloid A and other acute phase reactants to monitor the inflammatory response after castration in horses: a field study. Equine Vet J. 2005;37: 552–556. 16295934 10.2746/042516405775314853

[8] Hobo S, Niwa H, Anzai T. Evaluation of serum amyloid A and surfactant protein D in sera for identification of the clinical condition of horses with bacterial pneumonia. J Vet Med Sci. 2007;69: 827–830. 17827889 10.1292/jvms.69.827

[9] Dyson PK, Jackson BF, Pfeiffer DU, Price JS. Days lost from training by two- and three-year-old Thoroughbred horses: a survey of seven UK training yards. Equine Vet J. 2008;40: 650–657. 19165934 10.2746/042516408x363242

[10] Jackson BF, Lonnell C, Verheyen KLP, Dyson P, Pfeiffer DU, Price JS. Biochemical markers of bone metabolism and risk of dorsal metacarpal disease in 2-year-old Thoroughbreds. Equine Vet J. 2005;37: 87–91. 15651741 10.2746/0425164054406775

[11] Jackson BF, Dyson PK, Lonnell C, Verheyen KLP, Pfeiffer DU, Price JS. Bone biomarkers and risk of fracture in two- and three-year-old Thoroughbreds. Equine Vet J. 2009;41: 410–413. 19562906 10.2746/042516409x416206

[12] Verwilghen D, Busoni V, Gangl M, Franck T, Lejeune JP, Vanderheyden L, et al Relationship between biochemical markers and radiographic scores in the evaluation of the osteoarticular status of Warmblood stallions. Res Vet Sci. 2009;87: 319–328. 10.1016/j.rvsc.2009.02.002 19298987

[13] Turlo A, Cywinska A, Czopowicz M, Witkowski L, Szarska E, Winnicka A. Post-exercise dynamics of serum amyloid A blood concentration in thoroughbred horses classified as injured and non-injured after the race. Res Vet Sci. 2015;100: 223–225. 10.1016/j.rvsc.2015.04.008 25933933

[14] Cywińska A, Szarska E, Górecka R, Witkowski L, Hecold M, Bereznowski A, et al Acute phase protein concentrations after limited distance and long distance endurance rides in horses. Res Vet Sci. 2012;93: 1402–1406. 10.1016/j.rvsc.2012.02.008 22390917

[15] Cywinska A, Witkowski L, Szarska E, Schollenberger A, Winnicka A. Serum amyloid A (SAA) concentration after training sessions in Arabian race and endurance horses. BMC Vet Res. 2013;9: 91 10.1186/1746-6148-9-91 23634727 PMC3655847

[16] Pollock PJ, Prendergast M, Schumacher J, Bellenger CR. Effects of surgery on the acute phase response in clinically normal and diseased horses. Vet Rec. 2005;156: 538–542. 15849343 10.1136/vr.156.17.538

[17] EL-Deeb WM, El-Bahr SM. Selected biochemical indicators of equine rhabdomyolysis in arabian horses: acute phase proteins and trace elements. J Equine Vet Sci. 2014;34: 484–488.

[18] Zar JH. Biostatistical analysis 5th ed. Upper Saddle River: Prentice Hall; 2010.

[19] Hinchcliff KW, Kaneps AJ, Raymond JG. Equine sports medicine and surgery St. Louis: Elsevier Ltd; 2004.

[20] Wilsher S, Allen WR, Wood JLN. Factors associated with failure of thoroughbred horses to train and race. Equine Vet J. 2006;38: 113–118. 16536379 10.2746/042516406776563305

[21] Vandenplas ML, Moore JN, Barton MH, Ruossel AJ, Cohen ND. Concentrations of serum amyloid A and lipopolysaccharide-binding protein in horses with colic. Am J Vet Res. 2005;66: 1509–1516. 16261823 10.2460/ajvr.2005.66.1509

[22] El-Deeb WM, El-Bahr SM. Investigation of selected biochemical indicators of equine rhabdomyolysis in arabian horses: pro-inflammatory cytokines and oxidative stress markers. Vet Res Commun. 2010;34: 677–689. 10.1007/s11259-010-9439-5 20830520

[23] Jonsdottir IH, Schjerling P, Ostrowski K, Asp S, Richter EA, Pedersen BK. Muscle contractions induce interleukin-6 mRNA production in rat skeletal muscles. J Physiol. 2000;528 Pt 1: 157–163. 11018114 10.1111/j.1469-7793.2000.00157.x PMC2270126

[24] Steensberg A, van Hall G, Osada T, Sacchetti M, Saltin B, Klarlund Pedersen B. Production of interleukin-6 in contracting human skeletal muscles can account for the exercise-induced increase in plasma interleukin-6. J Physiol. 2000;529: 237–242. 11080265 10.1111/j.1469-7793.2000.00237.x PMC2270169

[25] Liburt NR, Adams AA, Betancourt A, Horohov DW, McKeever KH. Exercise-induced increases in inflammatory cytokines in muscle and blood of horses. Equine Vet J. 2010;42: 280–288. 10.1111/j.2042-3306.2010.00275.x 21059019

[26] Lamprecht ED, Bagnell CA, Williams CA. Inflammatory responses to three modes of intense exercise in Standardbred mares–a pilot study. Comp Exerc Physiol. 2009;5: 115.

[27] Donovan DC, Jackson CA, Colahan PT, Norton N, Hurley DJ. Exercise-induced alterations in pro-inflammatory cytokines and prostaglandin F2α in horses. Vet Immunol Immunopathol. 2007;118: 263–269. 17617470 10.1016/j.vetimm.2007.05.015

[28] Pedersen BK, Steensberg A, Fischer C, Keller C, Ostrowski K, Schjerling P. Exercise and cytokines with particular focus on muscle-derived IL-6. Exerc Immunol Rev. 2001;7: 18–31. 11579746

[29] Louis E, Raue U, Yang Y, Jemiolo B, Trappe S. Time course of proteolytic, cytokine, and myostatin gene expression after acute exercise in human skeletal muscle. J Appl Physiol. 2007;103: 1744–1751. 17823296 10.1152/japplphysiol.00679.2007

[30] Nieman DC, Henson DA, Smith LL, Utter AC, Vinci DM, Davis JM, et al Cytokine changes after a marathon race. J Appl Physiol (1985). 2001;91: 109–114. 11408420 10.1152/jappl.2001.91.1.109

[31] Horohov DW, Sinatra ST, Chopra RK, Jankowitz S, Betancourt A, Bloomer RJ. The effect of exercise and nutritional supplementation on proinflammatory cytokine expression in young racehorses during training. J Equine Vet Sci. 2012;32: 805–815.

[32] Nieman DC, Dumke CL, Henson DA, McAnulty SR, Gross SJ, Lind RH. Muscle damage is linked to cytokine changes following a 160-km race. Brain Behav Immun. 2005;19: 398–403. 16061149 10.1016/j.bbi.2005.03.008

[33] Millar NL, Wei AQ, Molloy TJ, Bonar F, Murrell GAC. Cytokines and apoptosis in supraspinatus tendinopathy. 2009;91: 417–424. 10.1302/0301-620X.91B3.21652 19258623

[34] Millar NL, Reilly JH, Kerr SC, Campbell AL, Little KJ, Leach WJ, et al. Hypoxia: a critical regulator of early human tendinopathy. 2012; 302–310. 10.1136/ard.2011.154229 21972243

[35] John T, Lodka D, Kohl B, Ertel W, Jammrath J, Conrad C, et al Effect of pro-inflammatory and immunoregulatory cytokines on human tenocytes. J Orthop Res. 2010;28: 1071–1077. 10.1002/jor.21079 20127972

[36] Berglund M, Hart DA, Wiig M. The inflammatory response and hyaluronan synthases in the rabbit flexor tendon and tendon sheath following injury. J Hand Surg Eur Vol. 2007;32: 581–587. 17950228 10.1016/J.JHSE.2007.05.017

[37] Hosaka Y, Kirisawa R, Yamamoto E, Ueda H, Iwai H, Takehana K. Localization of cytokines in tendinocytes of the superficial digital flexor tendon in the horse. J Vet Med Sci. 2002;64: 945–947. 12419874 10.1292/jvms.64.945

[38] Langberg H, Olesen JL, Gemmer C, Kjaer M. Substantial elevation of interleukin-6 concentration in peritendinous tissue, in contrast to muscle, following prolonged exercise in humans. J Physiol. 2002;542: 985–990. 12154195 10.1113/jphysiol.2002.019141 PMC2290459

[39] Jacobsen S, Niewold TA, Halling-Thomsen M, Nanni S, Olsen E, Lindegaard C, et al Serum amyloid A isoforms in serum and synovial fluid in horses with lipopolysaccharide-induced arthritis. Vet Immunol Immunopathol. 2006;110: 325–330. 16337010 10.1016/j.vetimm.2005.10.012

[40] Hultén C, Grönlund U, Hirvonen J, Tulamo R-M, Suominen MM, Marhaug G, et al Dynamics in serum of the inflammatory markers serum amyloid A (SAA), haptoglobin, fibrinogen and alpha2-globulins during induced noninfectious arthritis in the horse. Equine Vet J. 2002;34: 699–704. 12455841 10.2746/042516402776250405

[41] Jacobsen S, Thomsen MH, Nanni S. Concentrations of serum amyloid A in serum and synovial fluid from healthy horses and horses with joint disease. Am J Vet Res. 2006;67: 1738–1742. 17014325 10.2460/ajvr.67.10.1738

[42] Bauzá G, Miller G, Kaseje N, Wigner NA, Wang Z, Gerstenfeld LC, et al The effects of injury magnitude on the kinetics of the acute phase response. J Trauma. 2011;70: 948–953. 10.1097/TA.0b013e3181e1d27b 20693926 PMC3563334

[43] Buttenschoen K, Fleischmann W, Haupt U, Kinzl L, Buttenschoen DC. Translocation of endotoxin and acute-phase proteins in malleolar fractures. J Trauma. 2000;48: 241–245. 10697081 10.1097/00005373-200002000-00008

[44] Yoon SI, Lim SS, Rha JD, Kim YH, Kang JS, Baek GH, et al The C-reactive protein (CRP) in patients with long bone fractures and after arthroplasty. Int Orthop. 1993;17: 198–201. 8340178 10.1007/BF00186386

[45] Billinghurst RC, Fretz PB, Gordon JR. Induction of intra-articular tumour necrosis factor during acute inflammatory responses in equine arthritis. Equine Vet J. 1995;27: 208–216. 7556048 10.1111/j.2042-3306.1995.tb03064.x

[46] Ley C, Ekman S, Elmén A, Nilsson G, Eloranta ML. Interleukin-6 and tumour necrosis factor in synovial fluid from horses with carpal joint pathology. J Vet Med A Physiol Pathol Clin Med. 2007;54: 346–351. 17718806 10.1111/j.1439-0442.2007.00956.x

[47] Campbell MD, Bellamy JE, Searcy GP. Determination of plasma fibrinogen concentration in the horse. Am J Vet Res. 1981;42: 100–104. 7224302

[48] Jacobsen S, Nielsen JV, Kjelgaard-Hansen M, Toelboell T, Fjeldborg J, Halling-Thomsen M, et al Acute phase response to surgery of varying intensity in horses: a preliminary study. Vet Surg. 2009;38: 762–769. 10.1111/j.1532-950X.2009.00564.x 19674420

